# Toward a conceptual framework of health and its operational definition: an application in the 1958 British birth cohort

**DOI:** 10.1186/s12889-022-14967-z

**Published:** 2023-01-14

**Authors:** Camille Joannès, Hélène Colineaux, Gregory Guernec, Raphaële Castagné, Michelle Kelly-Irving

**Affiliations:** 1 Equity Research Team, CERPOP, Université de Toulouse, Inserm, Université Paul Sabatier, Toulouse, France; 2 Interdisciplinary Federal Research Institute On Health & Society (IFERISS), Université Toulouse III Paul Sabatier, Toulouse, France

**Keywords:** Resources, Reserves, Life course epidemiology, Adaptation

## Abstract

**Background:**

Defining and measuring Health presents a challenge, partly due to its conceptual pluralism. To measure Health as an ability to adapt and self-manage, we developed an approach within the theoretical framework of resources and reserves over the life course, recently proposed in the literature. We aimed to (i) use the conceptual framework developed to identify indicators of deteriorating health reserves, (ii) construct an overall health measure from these indicators, (iii) evaluate the association between the overall health measure and subsequent health outcomes and (iv) assess the robustness of our method.

**Methods:**

We used data from 7,043 individuals born in 1958 in Great Britain included in the National Child Development Study. An overall health measure was constructed via the sum of three selected indicators of deteriorating health reserves in mid-life: chronic widespread pain (CWP), Clinical Interview Schedule - revised (CIS-r), and allostatic load (AL). A three-category variable was defined: impaired/medium/optimal overall health. We explored criterion validity by modelling the relationships between the overall health measure, or each reserve taken separately at 44–45 years, and self-rated health at 46 years and mortality up to 58 years, corresponding to 14 years of follow up, using Cox and logistic regressions respectively. We performed comparative analyses to assess the robustness of the method.

**Results:**

Having an impaired overall health measure was significantly associated with all-cause premature mortality (HR_impaired_ = 2.74 [1.86; 4.05]) and an increased risk of later fair/poor/very poor self-rated health (OR_impaired_ = 7.50 [6.29; 8.95]). The overall health measure had a greater effect on the self-rated health estimates than each indicator of deteriorating health reserves considered separately (OR_AL medium_ = 1.82 [1.59; 2.09]; OR_AL high_ = 2.74 [2.37; 3.16]; OR_CIS-r_ = 5.20 [4.45; 6.08]; OR_CWP_ = 2.85 [2.53; 3.21]). CIS-r and allostatic load were also associated with premature mortality contrary to chronic widespread pain (HR_AL medium_1.82 [1.27; 2.61]; HR_AL high_ = 3.10 [2.19; 4.40]; HR_CIS-r_ = 1.77 [1.22; 2.56]; HR_CWP_ = 1.32 [0.98; 1.76]). The multiple comparative analyses conducted allowed us to assess the robustness of our method within this cohort.

**Conclusions:**

We proposed a method for measuring Health in mid-life in line with the concept of Health as the ability to adapt and self-manage and the concept of health reserves. This method may be applied and further developed within the field of social and positive epidemiology.

**Supplementary Information:**

The online version contains supplementary material available at 10.1186/s12889-022-14967-z.

## Introduction

Defining and measuring Health is complex due to its conceptual pluralism. Different models in different historical periods, have attempted to define the concept of Health [1]. The WHO definition of Health as “a state of complete physical, mental and social well-being and not merely the absence of disease or infirmity” [2], has an aspirational aim to guide national and global health governance. However, this definition does not provide a conceptual framework for the operationalisation of Health for Epidemiology, being limited by its utopian and unmeasurable nature [3, 4]. Since the Ottawa charter [5], the idea that Health should be seen as the ability to cope has grown increasingly popular in recent years. An alternative definition of Health has been proposed as the “ability to adapt and self-manage in the face of social, physical and emotional challenges” [3]. This definition means that Health can be considered as a dynamic rather than a static state, adding a temporal dimension. However, this definition does not consider the fact that this capacity for "adaptation and self-management" may differ among individuals depending on social, political, economic and environmental factors. Additional concepts are needed to address this limitation.

The longitudinal approach proposed by the life course theory considers the health of individuals as trajectories that evolve continuously following human development throughout the life span [6]. Health trajectories may represent a decline, as can be expected with ageing, involving the progressive loss of functional and cognitive abilities, or they may indicate an improvement, in the case of recovery from illness and associated disability [7]. These individual health trajectories are shaped through the long term influences of biological and social mechanisms but also by individual resources [6]. Such resources whether biological, cognitive, emotional, economic or relational, are built and used over the life course for immediate use and direct effect to enable functioning in daily life [8].

Sufficient resources at a given period of development allow the maintenance of reserves in which they are grouped. Reserves, as containers of resources, are latent capacities with a protective function that can be used (or not) to adapt to and self-manage when faced with adverse life events [8]. Health reserves include physical, socioemotional, cognitive [9] and physiological reserves [10]. The physical reserve of bodily functions refers to the physical or functional capacity of an individual built up over the life course to carry out activities successfully. It aims to maintain functional integrity and contributes to an adaptive response to environmental physical challenges [9]. The socioemotional reserve reflects the interaction between an individual’s psychological state and their surrounding social world. It characterises the ability, built up over a lifetime and influenced by social and cultural factors, to cope with an emotional and social stressor [10, 11]. The cognitive reserve corresponds to “the level of protection against clinical manifestations of neurological damage” i.e. the accumulated capacity throughout the life course to maintain and improve cognitive functioning and to delay its decline due to ageing [12]. The physiological reserve relates to the concept of allostasis, an active process where the body attempts to maintain normal physiological regulation in response to stress exposure [13]. The level of resources or the functioning of the reserves that individuals can rely upon may influence their ability to cope, with responses ranging between resilience and vulnerability. Vulnerability corresponds to a lack of resources or the reduced capacity of an individual to restore reserves that puts individuals at risk of experiencing the negative consequences of a stress or traumatic event followed by an inability to cope effectively and to recover [14]. The concept of reserves is still under development, and therefore subject to change in the light of new scientific findings. Taking into consideration the constitution, maintenance and activation of reserves over the life course may help researchers in field such as social epidemiology to identify factors that may affect the “ability to adapt and self-manage”.

The operationalisation of the health reserve concept presented above then leads to the following question: how can we measure the ability to adapt and self-manage i.e. the capacity of an individual to maintain or restore reserves? Since an impaired health trajectory is reflected by a chronically low level of resources which could have a cost on the health reserves, an optimal health trajectory with coping and adaptation can be understood as a non-deterioration of health reserves. Therefore, the results of a health trajectory, whether impaired or optimal, can be measured by identifying indicators of deteriorating health reserves, which by their occurrence could reflect an accommodation, in the Frisancho sense of compromised adaptation [15].

The identification of these indicators of deteriorating health reserves is challenging and may vary over time, across countries and be context specific. There is a need for a guide to operationalize Health with an approach to move from concept-to-method when measuring overall Health, applicable across epidemiological studies. This research will focus on the operationalization of Health in mid-life to consider Health as the result of a life trajectory and to measure it before the physical decline that occurs with aging. The identification of indicators of deteriorating health reserves to measure Health from a longitudinal trajectory perspective can ideally involve repeated measures of these indicators. However, this type of data is not always available. An alternative approach would be to identify different indicators of chronic deterioration of health states, each indicator referring to a health reserve. This indicator would correspond to an unfavorable health condition, at the stage of a clinical pathological manifestation with a consequent risk of a continuous and negative evolution over time. Based on the theoretical framework developed previously, a non-exhaustive list of indicators of deteriorating health reserves that could be available in epidemiological studies are listed in Table 1.

**Table 1:** Examples of indicators of deteriorating health reserves

• The physical reserve: indicators that can be used in practice to capture physical deterioration, i.e. a disabling condition affecting the mobility of the body, can include chronic pain, grip strength or physical mobility tests
• The socioemotional reserve: proxy variables [16] of socioemotional deterioration are including Clinical Interview Schedule - Revised (CIS-r) [17], Malaise inventory [18] or other validated screening instrument assessing psychological distress
• The cognitive reserve: the Trail Making Scale or other cognitive assessment can reveal deterioration in cognitive reserve
• The physiological reserve: to measure physiological deterioration or a deregulation of allostasis, allostatic load (AL) [19] or other biological scores [20] have been developed to measure the consequence of a prolonged activation of the stress response system by external challenges, leading to physiological imbalances across systems

We hypothesise that indicators of deteriorating health reserves in midlife, before the natural decline phase of later life, reflect the consequences of an accommodation of different health reserves over the life course and can be combined to measure Health.

The main objective of this study is to propose a standardised method for measuring overall Health by (i) using the theoretical framework developed to identify indicators of deteriorating health reserves, (ii) constructing an overall health measure from these indicators, (iii) evaluating the association between the overall health measure, each indicator and subsequent health outcomes and (iv) assessing the robustness of our method and the proof of concept within this cohort through comparative analyses.

## Methods

### Study population and follow-up

We used data from the 1958 National Child Development Study (NCDS 58), a birth cohort study including all people born in Great Britain during one week in 1958 (*n* = 18,555). Data collection on multiple aspects of the cohort members' lives, including health indicators, has been carried out from birth to age 60 by the Centre for Longitudinal Studies. The NCDS 58 has been described in detail elsewhere [21]. In 2002–2004 when individuals were aged 44–45 years, information was collected through a biomedical survey (blood, saliva samples and anthropometric measurements) and a home-based clinical assessment, with data available for 9,377 individuals. The selection of this wave of data at 44–45 years was relevant to conduct this study because it is the only wave in which both clinical and biomedical information were collected to derive indicators of deteriorating health reserves and the measure of overall health. Participants in this survey were found representative of the general cohort [22]. Participants' data were then selected at age 46 years to measure their self-rated health and up to age 58 years to measure mortality, which corresponds to 14 years of follow-up. A total of 2,334 participants were excluded from our analyses including pregnant women and those from whom blood was not obtained, as well as those with no data for the selected health reserve indicators and health outcomes, leaving a study sample of 7,043 participants (75% of the initial sample of the biomedical survey). The sample selection strategy is described in Additional Figure 1.

### Indicators of deteriorating health reserves

We first identified measurable items at 44/45y related to physical, psychosocial and physiological reserves to use them as indicators of deteriorating health reserves. No data on the deterioration of the cognitive reserve was available at this age.

#### Physical reserve

Chronic widespread Pain (CWP) is a common chronic pain measure used in several studies [23, 24] and is defined according to the American College of Rheumatology Criteria for the Classification of Fibromyalgia [25]: pain present for three months or longer, both above and below the waist; on both the left and right sides of the body; and in the axial skeleton. NCDS participants were asked ‘‘During the past month, have you had any ache or pain which has lasted for one day or longer?”. Those who answered positively were asked to indicate the location of their pain(s) on a four-view body manikin, and to indicate whether they had been aware of the pain for more than three months. Using this data, we constructed a binary variable identifying participants with CWP.

#### Socioemotional reserve

We used the revised CIS-r where scores of ≥ 12 indicate common mental disorders [17]. In the NCDS 58, an abbreviated version of the CIS-r was used (sections enquiring after worry, obsessions, somatic symptoms, compulsions and physical health worries were omitted) [26]. The domains that constitute this score in the NCDS cohort are: fatigue, concentration and forgetfulness, sleep problems, irritability, depression, suicidal ideation, anxiety, phobias and panic. Each domain provides a score from 0 to 4 (or 0 to 5 for suicidal ideation) based on the sum of their related items. The sum of these 14 domains resulted in an overall CIS-r score ranging from 0 to 33. We categorised this score into two groups “No mental health problem/Common mental health problem” according to the cut-off of 9 adapted from the abbreviated version of the CIS-r of the NCDS 58 [27].

#### Physiological reserve

We used data from the biomedical survey to construct an allostatic load score. Based on previous work carried out within the NCDS 58 [28], four physiological systems have been identified with the following 14 available and related biomarkers: the neuroendocrine system (salivary cortisol t1, salivary cortisol t1–t2); the immune and inflammatory system (insulin-like growth factor-1 (IGF1), C-reactive protein (CRP), fibrinogen, Immunoglobulin E (IgE)); the metabolic system (high-density lipoprotein (HDL), low-density lipoprotein (LDL), triglycerides, glycosylated hemoglobin (HbA1C)); the cardiovascular and respiratory systems: (systolic blood pressure (SBP), diastolic blood pressure (DBP), heart rate, peak expiratory flow). Using sex-specific quartiles, each biomarker was dichotomized into "high" (coded as 1) and "low" (coded as 0) risk. The sum of these 14 dichotomized biomarkers resulted in an overall AL score ranging from 0 to 14 where a higher score represented a high AL. This AL was also recoded into a 3 category variable based on tertiles in the sample, where a score of 0–2 was considered to be “low”, 3–4 as “middle”, and 5–14 as “high” [29].

### Overall health measure

We constructed the *overall health measure* variable using the selected indicators of deteriorating health reserves: CWP (2 categories), CIS-r (2 categories) and AL (3 categories). With regard to the theory previously developed, we supposed that each indicator of deteriorating health reserves has the same weight as the others. To ensure equal weighting between these variables, so that each variable varies from 0 to 1, we first reformatted AL to ensure that its number of categories (*n* = 3) varied from 0 to 1. We rescaled its first category to 0, its second to 0.5 and its third to 1. Second, we summed AL, CWP and CIS-r. We finally classified our overall health measure into three groups “optimal [0]/medium[0.5–1]/impaired[1.5–3]”.

### Health outcomes: Self-rated health and mortality

To assess our overall health measure, we selected two later health outcomes: mortality and self-rated health since health refers not only to being alive but also to living well [30].

All-cause mortality was derived from death certificates from the National Health Service Central Register recorded by the Centre for Longitudinal Studies. The mortality data most recently available to researchers provided information on date of death up to December 2016. The 14-year follow-up was calculated from the date of blood collection to the date of death. Individuals with no death data but who had others data between the ages of 44/45 and 60 were classified as alive.

Self-rated health (SRH) was created using cohort members’ responses at 46y to the question “How would you describe your health generally?”. Responses were dichotomised into “excellent/good” versus “fair/poor/very poor”. In order to maximise the statistical power of our study sample, individuals with missing data at age 46 but who reported excellent/good perceived health at 50y and 55y (*n* = 580) were classified in the good self-rated health group at age 46 and those reporting fair/poor/very poor self-rated health at 50y and 55y (*n* = 138) were classified in the poor self-rated health group at age 46.

### Statistical analysis

The content and construct validity of the overall health measure are ensured by its relevance to the theoretical framework developed in the introduction section. We explored criterion validity of the overall health measure by modelling the relationship between indicators of deteriorating health reserves, the overall health measure and later health outcomes (i.e. mortality and SRH) [9]. First, descriptive and bivariate statistics were carried-out to estimate the association of our selected indicators of deteriorating health reserves variables with self-rated health and mortality outcomes, using Chi square or logrank tests [31] as appropriate. Kaplan–Meier curves [32] were draw for participants’ indicators of deteriorating health reserves and participants’ overall health measure to validate the application conditions of the logrank tests. Second, associations between each of the indicators of deteriorating health reserves and the overall health measure with two later health outcomes were modelled using logistic regressions models for self-rated health and using multivariate Cox proportional regression [33] for mortality. Odds-ratio (OR) with 99% confidence intervals (CI) and hazard ratios (HR) with 99% CI were reported for the logistic regression models and Cox proportional regression respectively. We tested the proportional hazards assumption for each indicator of deteriorating health reserves and for the overall health measure. Both statistical testing using Schoenfeld residuals [34] and visual inspection (scatterplots of scaled Schoenfeld residuals vs. time) were performed and showed no violation of the proportional hazards assumption. Because perceived health and mortality differ between women and men [35, 36], all models were adjusted for sex. We also estimated the sensitivities and specificities of the overall health measure in relation to mortality and self-rated health.

### Comparative analyses

We conducted comparative analyses to assess the robustness of the method [37] and to assess the proof of concept. To ensure that the results observed were not biased by the grouping of the overall health measure, we tested an alternative grouping of the overall health measure, combining the 0 and 0.5 categories, and we performed the same multivariate analyses with the health outcomes (i.e. mortality and SRH). To ensure that the results observed were not biased by the variables constituting the overall health measure, we constructed other overall health measures with a different categorisation of variables constituting them (overall health measures n°2 and n°3) or with a different choice of variables (overall health measure n°4) and we performed the same multivariate analyses with the health outcomes. A detailed description of the construction of the overall health measures n°2, 3 and 4 and their respective variables is given in Additional File 1. To ensure that the results observed were not biased by the data of the sample, we generated two sub-samples using a negative control variable (or neutral variable) to perform multivariate analyses and assess the association between the overall health measure with the health outcomes. In the first sample, we selected all individuals who responded to the biomedical survey in odd-numbered months, and the second sample included all individuals who responded to the questionnaire in even-numbered months. We hypothesised that the trend of the results would be similar between the two sub-samples. We also carried out other comparative analyses to ensure that the estimates obtained of our health reserve indicators on self-rated health at age 46 did not differ at ages 50 and 55 (See Additional Figure 2).

All statistical analyses were performed using Stata ®v17 software [38].

## Results

A detailed description of the study population is given in Table 2. Among the 7,043 included participants with 14 years of follow up, around 23% of them had a high allostatic load, 28% had chronic widespread pain and 11% had a common mental health problem at 44/45y. Two years later, 22% described their health as fair/poor/very poor at 46y and about 3% died between 44 and 58y.

**Table 2:** Descriptive analysis on the NCDS 58 sample (*n* = 7,043)

Variable	Level	n (%)
**Allostatic load 44/45y**	Low	3,162 (44.90)
	Medium	2,267 (32.19)
	High	1,614 (22.92)
**CWP 44/45y**	No	5,052 (71.73)
	Yes	1,991 (28.27)
**CIS-r 44/45y**	No mental disorder	6,274 (89.08)
	Common mental disorders	769 (10.92)
**Self-rated health 46y**	Excellent/Good	5,526 (78.46)
	Fair/Poor/Very poor	1,517 (21.54)
**Mortality 44-58y**	Alive	6,843 (97.16)
	Dead	200 (2.84)
**Sex**	Men	3,433 (48.74)
	Women	3,610 (51.26)
**Total**		7,043 (100.00)

Table 3A presents the distribution of the overall health measure and Table 3B provides details on the constitutive categories by indicators of deteriorating health reserves. About one third of individuals had an optimal overall health measure, corresponding to a low allostatic load, no mental disorder and no chronic widespread pain. Half of the remaining individuals had a medium overall health measure, corresponding to the deterioration in one of the indicator of health reserve. The remaining group had deterioration in two or all three-health indicators.

**Table 3:** Overall health measure distribution on the NCDS 58 sample (*n* = 7,043)

A. Overall health measure			B. Corresponding indicators of deteriorating health reserves			
Level	n (%)	Value	AL	CIS-r	CWP	n (%)
**Optimal**	2,153 (30.57)	0	Low	No	No	2,153 (30.57)
**Medium**	3,395 (48.20)	0.5	Medium	No	No	1,524 (21.64)
		1	High	No	No	984 (13.97)
			Low	Yes	No	169 (2.40)
			Low	No	Yes	718 (10.19)
**Impaired**	1,495 (21.23)	1.5	Medium	Yes	No	126 (1.79)
			Medium	No	Yes	499 (7.09)
		2	High	Yes	No	96 (1.36)
			Low	Yes	Yes	122 (1.73)
			High	No	Yes	396 (5.62)
		2.5	Medium	Yes	Yes	118 (1.68)
		3	High	Yes	Yes	138 (1.96)
Total						7,043 (100.00)

### Criterion validity with mortality and SRH

Table 4 introduces the distribution of the selected indicators of deteriorating health reserves for NCDS 58 participants according to (A) probability of survival and (B) their self-rated health. From 3A, the probability of survival was highest in the low allostatic load group, and dropped gradually across groups with the high allostatic load group having the lowest probability of survival. Cohort members who reported chronic widespread pain and mental disorders had a lower probability of survival compared respectively to those with no pain and no mental disorders. From 3B, compared to participants who had excellent/good self-rated health, those who reported a fair/poor/very poor self-rated health were more likely to have had a medium or a high allostatic load and to have suffered from CWP and common mental disorders previously.

**Table 4:** Bivariate statistics of the distribution of (A) mortality and (B) self-rated health according to indicators of deteriorating health reserves variables on the NCDS 58 sample (*n* = 7,043)

		**(A) Mortality 44y-58y**			**(B) Self-rated health 46y**		
		**Survival probability at 58y**	**CI [95%]**	***p*-value(a)**	**Excellent/Good**	**Fair/Poor/Very poor**	***p*-value(b)**
					*n* = 5,357	*n* = 1,483	
					n (%)	n (%)	
**Allostatic load**	Low	0.984	[0.979; 0.987]	< 0,001	2,699 (48.84)	463 (30.52)	< 0,001
	Medium	0.970	[0.962; 0.976]		1,730 (31.31)	537 (35.40)	
	High	0.950	[0.939; 0.960]		1,097 (19.85)	517 (34.08)	
**CWP**	No	0.974	[0.969; 0.978]	0.0671	4,239 (76.71)	813 (53.59)	< 0,001
	Yes	0.966	[0.957; 0.973]		1,287 (23.29)	704 (46.41)	
**CIS-r**	No mental disorder	0.974	[0.969; 0.977]	0.005	5,365 (97.09)	1,265 (83.39)	< 0,001
	Common mental disorders	0.956	[0.939; 0.968]		161 (2.91)	252 (16.61)	

*P*-values were calculated using (a) a Log-rank test and (b) a chi-squared test

**Fig. 1:**
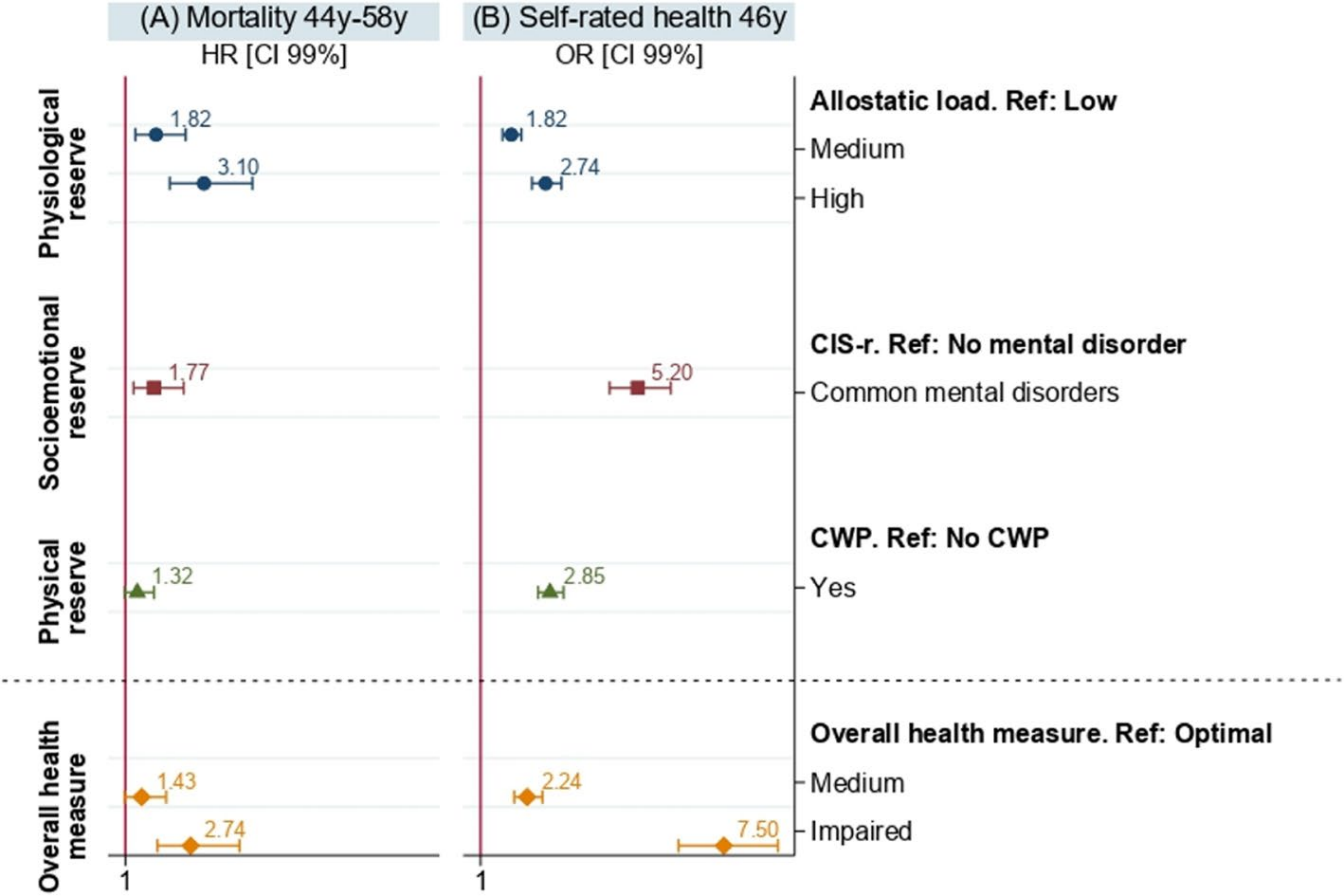
Sex adjusted results of Cox and Logistic regressions of the indicators of deteriorating health reserves and the overall health measure on (**A**) mortality and (**B**) self-rated health on the NCDS 58 sample (*n* = 7,043)

The results of the regressions models where each indicators of deteriorating health reserves and the overall health measure were analysed in relation to mortality and self-rated health are presented in Fig. 1A and B respectively. Participants with a medium or high allostatic load at 44/45 years or with a common mental disorder were significantly more at risk of death compared to participants with a low allostatic load or without a mental disorder (${HR}_{AL medium}$= 1.82 [1.27; 2.61]; ${HR}_{AL high}$= 3.10 [2.19; 4.40]. ${HR}_{CIS-r}$= 1.77 [1.22; 2.56]. Figure 1A). Individuals with an impaired overall health measure were significantly more at risk of death than those with an optimal overall health measure (${HR}_{impaired}$= 2.74 [1.86; 4.05]).

Similarly, participants with medium or high allostatic load at 44/45 years were more likely to report fair/poor/very poor self-rated health two years later (${0R}_{AL medium}$=1.82 [1.59; 2.09]; ${OR}_{AL high}$=2.74 [2.37; 3.16]), as well as those with mental disorders (${OR}_{CIS-r}$= 5.20 [4.45; 6.08]) and those with chronic widespread pain (${OR}_{CWP}$= 2.85 [2.53; 3.21]). Likewise, the probability of reporting fair/poor/very poor self-rated health was higher for participants with a medium overall health measure and even higher for those with an impaired overall health measure compared to those with an optimal overall health measure (${OR}_{medium}$= 2.24 [1.90; 2.65]; ${OR}_{impaired}$= 7.50 [6.29; 8.95]).

The results of the sensitivity (true positive rate) and specificity (true negative rate) analyses between each indicators of deteriorating health reserves and the overall health measure with mortality and self-rated health is presented in Table 5A and B respectively. Regarding the sensitivity analyses, 81% (95%CI = [74; 86]) of individuals who died between 44 and 58y and 86% (95%CI = [85; 88]) of individuals whose self-rated health was fair/poor/very poor, had a medium or impaired overall health measure. Regarding the specificity analyses, 31% (95%CI = [30; 32]) of individuals who did not die between 44 and 58y and 35% (95%CI = [34; 37]) of individuals whose self-rated health was excellent/good, had an optimal overall health measure.

**Table 5:** Sensitivity (Se) and Specificity (Spe) results on the NCDS 58 sample (*n* = 7,043)

		**(A) Mortality 44y-58y**				**(B) Self-rated health 46y**			
		Se	95% CI	Spe	95% CI	Se	95% CI	Spe	95% CI
AL	Low; Medium/High	74%	[67; 80]	45%	[44; 47]	70%	[67; 72]	49%	[48; 50]
CIS-r	No mental disorder; Common mental disorder	17%	[12; 23]	89%	[89; 90]	27%	[25; 29]	94%	[93; 94]
CWP	No; Yes	34%	[28; 41]	72%	[71; 73]	46%	[44; 49]	77%	[76; 78]
Overall health measure	Optimal; Medium/Impaired	81%	[74; 86]	31%	[30; 32]	86%	[85; 88]	35%	[34; 37]

### Robustness of the method

#### Regarding the grouping of the overall health measure

**Fig. 2:**
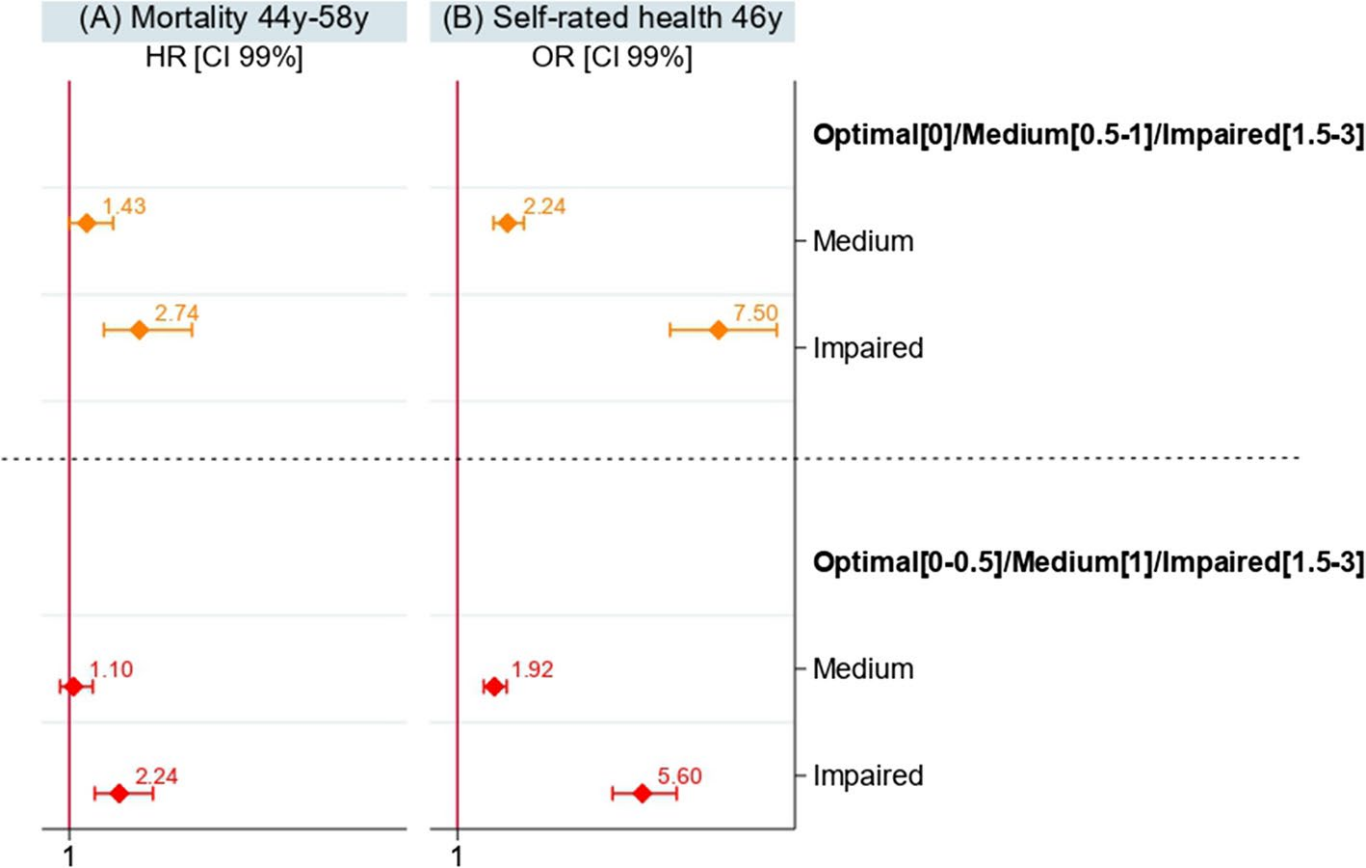
Sex adjusted results of Cox and Logistic regressions models of the two different categorisations of the overall health measure on (**A**) mortality and (**B**) self-rated health (*n* = 7,043)

Comparative analyses with the alternative grouping of the overall health measure, combining the 0 and 0.5 categories, are presented in Fig. 2 and showed similar effect patterns regardless of the overall health measure categorisation.

#### Regarding the variables constituting the overall health measure

**Fig. 3:**
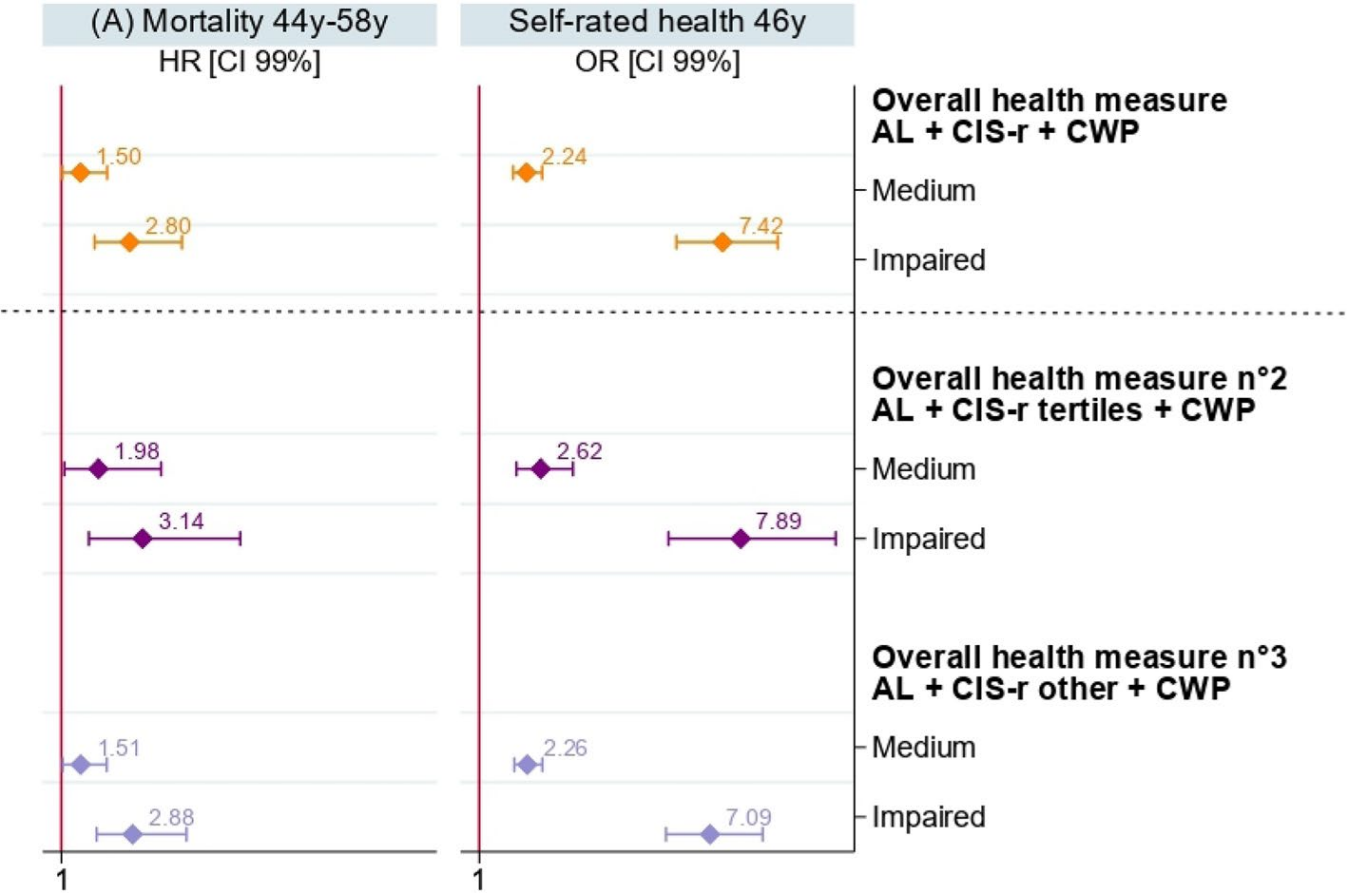
Sex adjusted results of Cox and Logistic regressions models of the overall health measure previously constructed and the overall health measures n°2 and 3 on (**A**) mortality and (**B**) self-rated health on the NCDS 58 sample (*n* = 7,043)

**Fig. 4:**
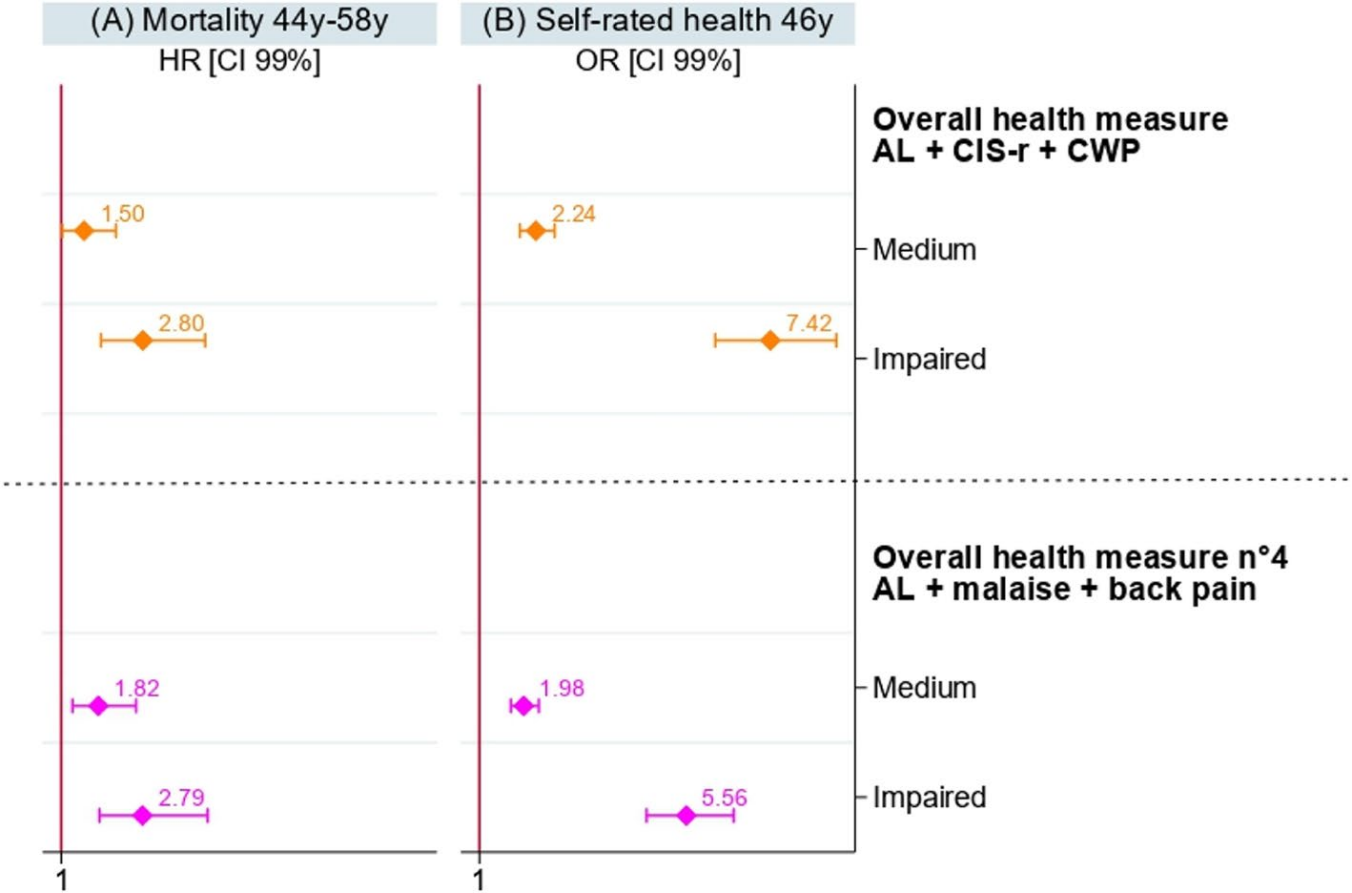
Sex adjusted results of Cox and Logistic regressions models of the overall health measure previously constructed and the overall health measure n 4 on (**A**) mortality and (**B**) self-rated health on the NCDS 58 subpopulation of complete case data (*n* = 6,820)

Comparative analyses with the overall health measures n°2, 3 and 4 and the previously constructed overall health measure on mortality and self-rated health are presented in Figs. 3 and 4. The trends observed are similar to the previous results despite a different categorisation of the CIS-r variable to construct these overall health measures and despite a different choice of items on another sweep of data within this cohort.

#### Regarding the data of the sample

**Fig. 5:**
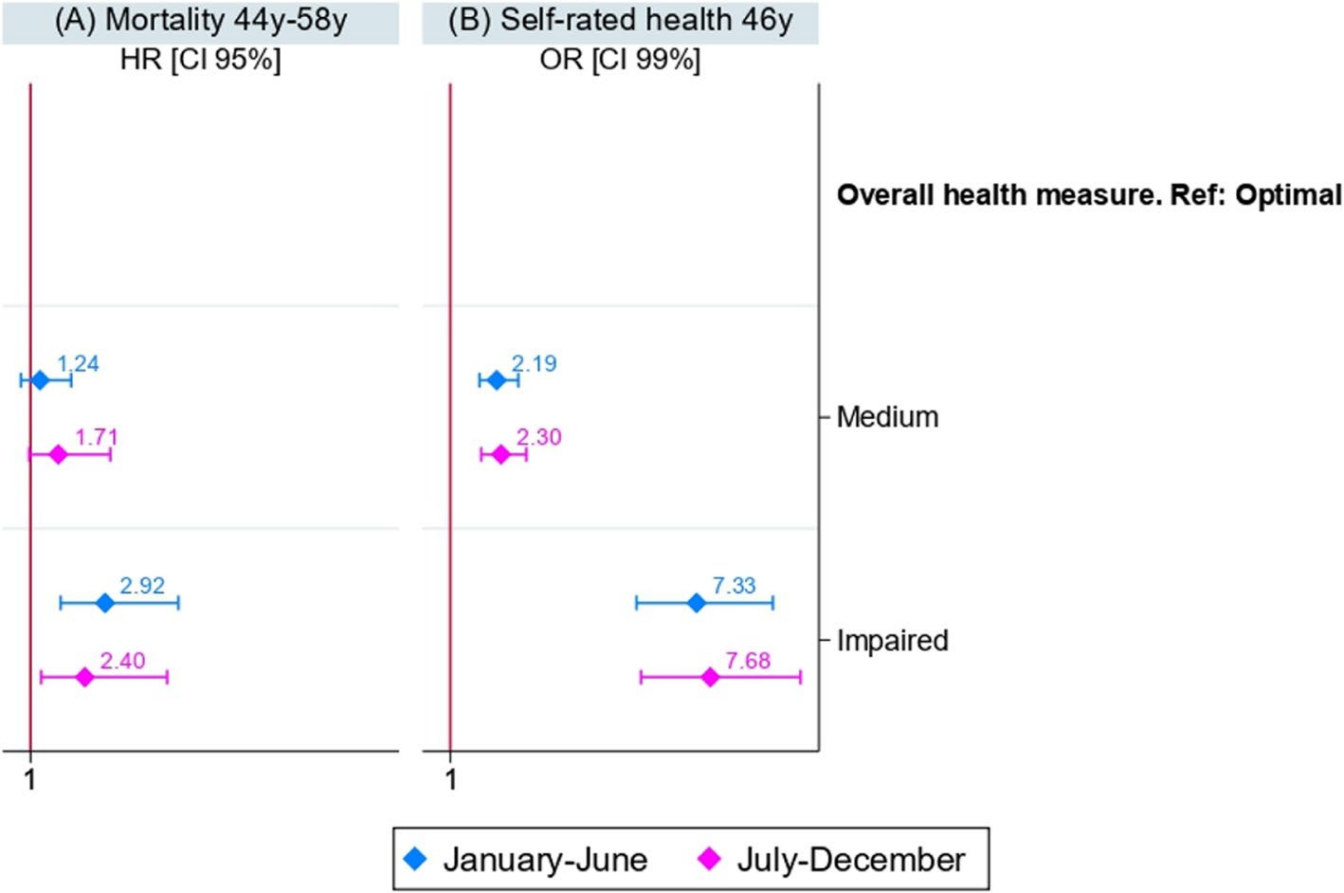
Sex adjusted results of Cox and Logistic regressions models of the overall health measure on (**A**) mortality and (**B**) self-rated health using a negative control variable

Analyse of the effect of a negative control variable (or neutral variable) on the association between the overall health measure and mortality and self-rated health are presented in Fig. 5. The trends observed of the OR and HR are similar regardless of the month of response to the survey.

## Discussion

Our study aimed to propose a standardised method to measure overall Health resulting from adaptation to conditions and circumstances across the life course by constructing and validating a concept-to-method approach. This overall health measure was obtained via the sum of indicators of deteriorating of physiological, physical and socioemotional health reserves, measured in a prospective UK birth cohort. As results, we notably found that one third of people had an optimal overall health measure at mid-life, half of the individuals had an average overall health measure while 20% had an impaired one.

To assess the criterion validity of this overall health measure, we analysed its association with two health outcomes, mortality and self-rated health. Our study suggests that having an impaired overall health measure was significantly associated with all-cause premature mortality over a 14-year period or an increased risk of fair/poor/very poor self-rated health two years later.

To examine to what extent each component of the overall health measure contributed to the increased risk of poor health outcomes, we also analysed the relationship between each of its indicators of deteriorating health reserves and mortality and self-rated health. Our analyses revealed that each indicator of deteriorating health reserves was related to self-rated health, consistent with results of previous studies [27, 39, 40]. However, the overall health measure had the greater effect on the self-rated health estimate than each indicator of deteriorating health reserves. These results suggest that the overall health measure provided a synthetic measure directly associated with self-rated health compared to each respective indicators of deteriorating health reserves analyzed separately.

Regarding mortality, both AL and CIS-r were related to premature mortality with AL having the strongest association compared to the other indicators of deteriorating health reserves. CWP was not associated with premature mortality. These findings are consistent with results of previous studies showing that AL [41–43], and mental disorder [44] were associated with all-cause premature mortality while CWP was mildly associated with an increased risk of death [45]. However, the overall health measure had smaller effect on the HR estimate than the AL variable probably due to the CWP variable constituting it and probably because AL is a measure of biology that is more likely to be related to mortality. However, given that Health is not defined solely by mortality and that the overall health measure is also related to self-rated health and more strongly than the allostatic load variable, these results assess that the overall health measure is able to measure Health not unidimensionally via mortality, but also with self-rated health.

To assess the robustness of the method, we conducted comparative analyses. We found that the results observed were not biased by the grouping of the overall health measure when we combined the 0 and 0.5 categories. We also found that the results observed were not biased by the variables constituting the overall health measure when we constructed other overall health measure with modified variable categories or when we used another set of variables to construct it. We also observed that the results were not biased by the data of the sample when we used a negative control variable. Finally, we found the same pattern of results even if we used self-rated health at other ages. This set of analyses validates this approach [37] providing a body of evidence for a proof of concept to measure of overall Health as the result of a process of adaptation over the life course.

The category of impaired health measured in our study raises questions on the concept of wear and tear, which can lead to damage predisposing the organism to disease. Wear and tear can be the result of a natural process associated with ageing, as well as the result of life course processes [11, 46] which may be an adaptation with a cost on the health reserves, i.e. an accommodation [15]. Indeed, this inability to recover from stress or to take advantage of recovery opportunities, within a given time frame, could lead to progressive and cumulative wear and tear, increasing the slope of decline of the health trajectory and the tipping point into vulnerability [8]. This concept of wear and tear, which is applied in the literature to one of the indicators used in our study, namely allostatic load [19], has been shown to predict the decline of health trajectories [47]. Health reserves and health wear and tear thus could appear to be dynamic mechanisms, interdependent and in constant dialogue. Future theoretical development is necessary to better understand the wear and tear mechanism with regard to the health reserve concept.

As a mirror to this measure of impaired health, there is the “optimal” category of the overall health measure [48]. This category may reflect an optimal adaptive health trajectory that mitigates the slope of decline. This implies that individuals with an optimal overall health measure may have benefitted from adaptive mechanisms optimising their health reserves and have had the means to recover from idiosyncratic adverse events. These adaptive mechanisms may be defined as an overall positive health mechanism that requires further investigation for its understanding.

The main weakness of this study is related to the selection of the items in our overall health measure due to data availability. Specifically, we were not able to use a measure of an indicator of deteriorating cognitive reserve. We did not weight the items before summing them to calculate the overall health measure, thus assuming that each health reserve played an equivalent role in the construction of the overall health measure. In addition, we have not weighted our indicators with a single health outcome, considering that Health we aim to measure could be multidimensional. The overall health measure, as well as the indicators, were used in a categorical format to separate groups of individuals sharing common characteristics and to identify those at greater risk of poor health. We rescaled the AL and CIS-r indicator into a categorical format to have the same format as CWP. It would have been interesting to use these indicators in a continuous format, which we were unable to do due to data availability. Other methods can be employed to measure overall health, using latent variables in principal component analysis for example. However, this alternative approach requires a panel of available variables associated with the concepts of health reserves with limitation regarding the interpretation of the results because of their variability. In addition, it would not allow a comparison between different groups with different health status. Overall our relatively simple approach, which uses indicators of deteriorating health reserves ranging from 0 to 1, ultimately creating a single sum score, has been shown in our study to successfully capture a common variance between its different components as we hypothesised. The proposed measure of health has low specificity with mortality and self-rated health, implying that it is not an accurate measure of optimal health. Further methodological research is needed to measure optimal health. The small size of the number of deaths (*n* = 200 or 2.84%) had reduced statistical power, and the association estimates were subsequently weakened, compared with what would have been estimated with a larger sample of individuals and consequently a larger number of deaths. However, their gradual distributions gave indications of underlying relationships. We measured Health at mid-life within this cohort due to the availability of data. Biomarkers were only collected at 44/45y and we used the latest release of mortality data in which deaths were reported up to 2016. We also chose to measure Health before the physical decline that occurs with aging. Additional research is needed to explore and validate other measures of Health at other ages. Further research is also needed to understand how social, political, economic, and environmental aspects may alter health reserves. This operationalization of Health could be effective in different populations with different health status, but this needs to be verified by conducting similar analyses in other cohorts. There is now a continuing need to compare the results of this study using the same method but in other cohorts.

## Conclusion

Using a UK national birth cohort study, multidimensional Health as the result of a life course adaptation process was measured using an overall health measure, combining indicators of deteriorating health reserves in mid-life, before the natural decline phase of later life. This conceptualization and operationalization of Health through the lens of coping and adaptation mechanisms reverses the traditional way of considering Health as the presence of diseases and/or intermediate phenotypes of diseases. While much attention is paid to the etiopathogenesis of the disease, with a detailed analysis of the semiology, syndromes, symptoms, and concepts of systemic exogenous or endogenous causes of the disease, this conceptualization and operationalization of Health can be applied in the field of social epidemiology for social inequalities in health research, with a needed development of the concept of wear and tear. This conceptualization and operationalization can also be applied in the field of positive epidemiology [49] which proposes a shift away from the traditional medical focus on people who are already ill to focus on what it means to flourish.

## Supplementary Information

**Additional file 1.**

## Data Availability

The datasets analysed during the current study are available in the UK Data Service repository, accessed upon registration be with UK-data-Archive (https://www.data-archive.ac.uk/) [50–54]. Administrative permissions were required to access the raw data from the UK Data Service and it was approved by the Centre for Longitudinal Studies (CLS) Data Access Committee team. All methods were carried out following relevant guidelines and regulations.

## Abbreviations

CIS-r: Clinical Interview Schedule - Revised
CWP: Chronic widespread Pain
AL: Allostatic load
NCDS 58: 1958 National Child Development Study
SRH: Self-rated health
Se: Sensibility
Spe: Specificity
OR: Odds-ratio
HR: Hazard ratios
CI: Confidence intervals
