# Supplementary material for: Toward a conceptual framework of health and its operational definition: an application in the 1958 British birth cohort
Source: BMC Public Health. 2023 Jan 14;23:100. doi: 10.1186/s12889-022-14967-z (PMC9840257; doi:10.1186/s12889-022-14967-z)
Supplement: Supplementary file 1 — Additional file 1. [file 12889_2022_14967_MOESM1_ESM.docx]

# Additional Figures

##
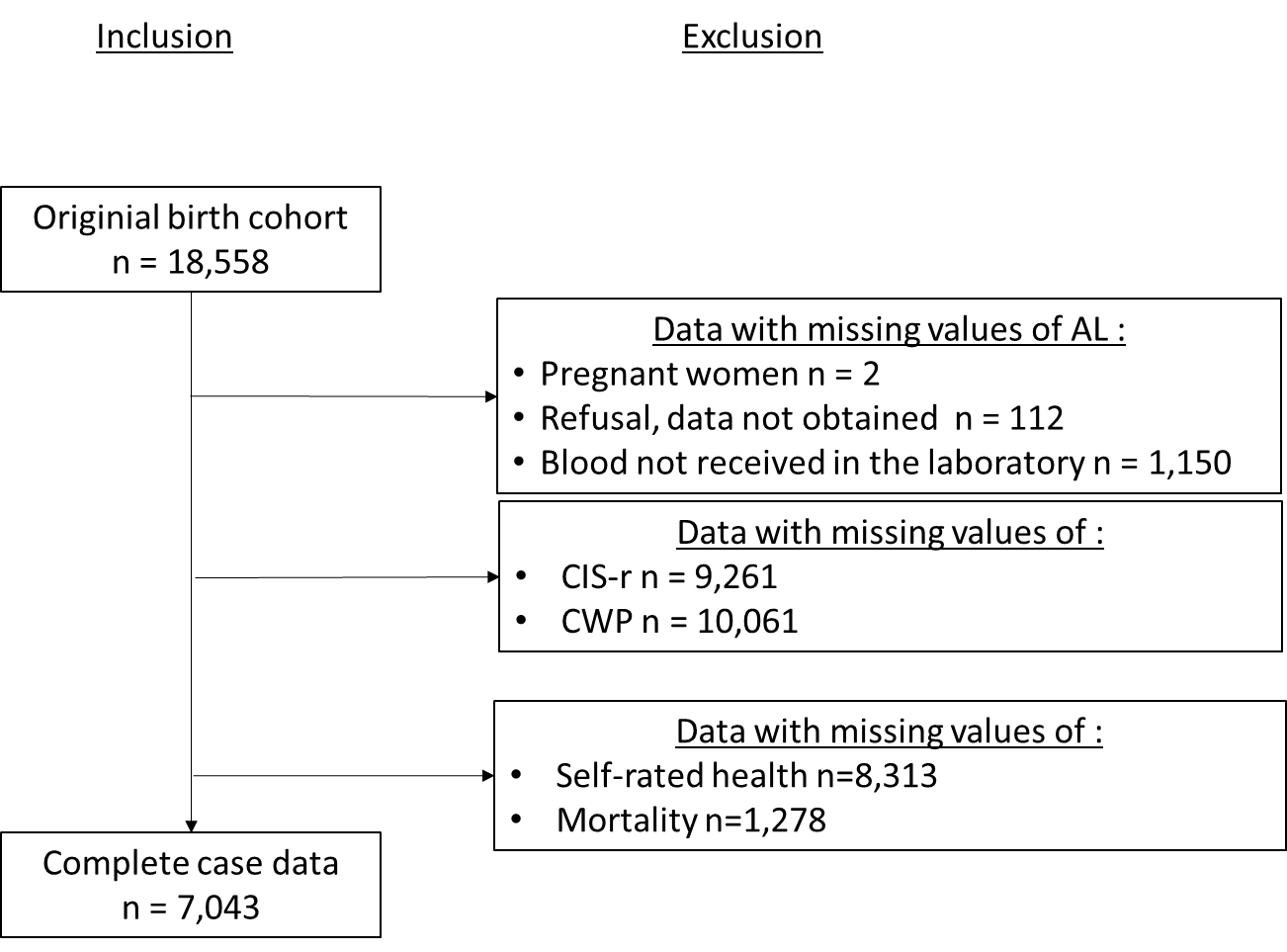
Additional Figure 1: Diagram of inclusion and exclusion criteria in the NCDS 58

## Additional Figure 2: Sex adjusted results of Logistic regressions of the indicators of deteriorating health reserves on self-rated health at ages 46, 50 and 55


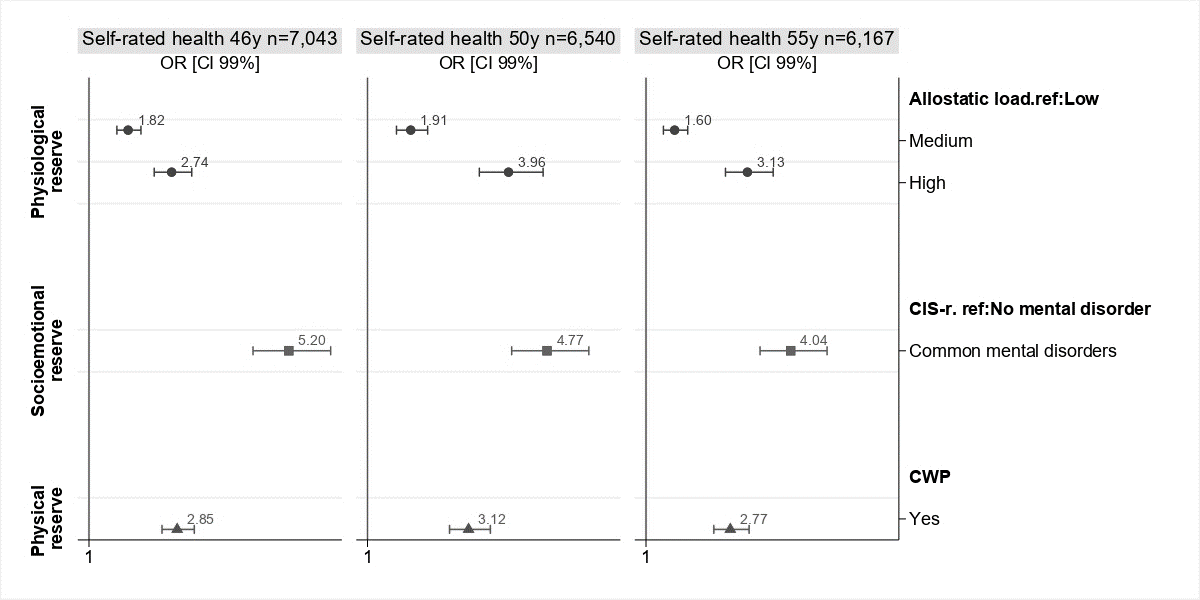


# Additional File 1. Details on the construction of the Global Health Score variables n°2, 3 and 4

- Overall health measure n°2: created from the same variables as the initial measure but with different categorization of the CIS-r variable

= AL/2 + CIS-r other categories + CWP

- Overall health measure n°3 = created from the same variables as the initial measure, but using the tertiles of the CIS-r variable for its categorization

= AL/2 + CIS-r tertiles+ CWP

- Overall health measure n°4: created from other variables than the initial measure except for the allostatic load which is maintained

= AL/2 + Malaise at 42y + Pain at 42y

| Overall health measure | NCDS sweep | Variable | Level | n(%) | Details |  |  |  |
| --- | --- | --- | --- | --- | --- | --- | --- | --- |
| n° 2 | Biomedical survey (44/45y) | CIS-r other categories | No mental health problem | 8,658 (46.65) | We have categorised the CIS-r score variable in the same method as in a previous article(1) | | | |
|  |  |  | Mental health problem requiring assessment | 438 (2.36) |  |  |  |  |
|  |  |  | Mental health problem requiring treatment | 201 (1.08) |  |  |  |  |
|  |  |  | Missing | 9,261 (49.90) |  |  |  |  |
|  |  |  |  |  |  |  |  |  |
| n° 3 | Biomedical survey (44/45y) | CIS-r tertiles | [0] | 3,381 (18.22) | We have categorised the CIS-r score variable using the tertiles of its distribution | | | |
|  |  |  | [1-4] | 3,380 (18.21) |  |  |  |  |
|  |  |  | [5-33] | 2,536 (13.67) |  |  |  |  |
|  |  |  | Missing | 9,261 (49.90) |  |  |  |  |
|  |  |  |  |  |  |  |  |  |
| n° 4 | Sweep n°6 (42y) | Pain at 42y | None | 8,834 (47.60) | Individuals were asked "Have you ever had or been told you had (i) persistent back pain lumbago or sciatica; (ii) chronic fatigue syndrome better known as ME" ; (iii) Neither of these". Those wo responded “Persistent back pain/lumbago/sciatic” were assigned to one group while the others were assigned to the reference category. | | | |
|  |  |  | Persistent back pain/lumbago/sciatic | 2,542 (13.70) |  |  |  |  |
|  |  |  | Missing | 7,182 (38.70) |  |  |  |  |
|  |  |  |  |  |  |  |  |  |
|  |  | Malaise inventory at 42y | Normal 0-7 | 9,767 (52.63) | Malaise is “a generalized feeling of discomfort, illness, uneasiness, fatigue or lack of well-being, often associated with a disease state” (2). We summed the binary malaise inventory variables to derive the malaise inventory score ranging from 0-24. Individuals responding ‘yes’ to eight or more of the 24 items are considered to be at risk of depression (3). | | | |
|  |  |  | Depressed 8-24 | 1,494 (8.05) |  |  |  |  |
|  |  |  | Missing | 7,297 (39.32) |  |  |  |  |
|  |  |  | Total | 18,558 (100) |  |  |  |  |

# References

1. Bebbington PE, McManus S. Revisiting the one in four: the prevalence of psychiatric disorder in the population of England 2000–2014. Br J Psychiatry. 2020 Jan;216(1):55–7.

2. Scully C. 4 - Signs and symptoms. In: Scully C, editor. Scully’s Medical Problems in Dentistry (Seventh Edition) [Internet]. Oxford: Churchill Livingstone; 2014. p. 97–122. Available from: https://www.sciencedirect.com/science/article/pii/B9780702054013000047

3. Rodgers B, Pickles A, Power C, Collishaw S, Maughan B. Validity of the Malaise Inventory in general population samples. Soc Psychiatry Psychiatr Epidemiol. 1999 Jun 25;34(6):333–41.
